# The impact of gender medicine on neonatology: the disadvantage of being male: a narrative review

**DOI:** 10.1186/s13052-023-01447-2

**Published:** 2023-06-06

**Authors:** Claudio Migliori, Marta Braga, Virginia Siragusa, Maria Cristina Villa, Livio Luzi

**Affiliations:** 1 Department of Neonatology, Ospedale San Giuseppe MultiMedica, 20123 Milan, Italy; 2 Department of Endocrinology, Nutrition and Metabolic Diseases, IRCCS MultiMedica, 20099 Sesto San Giovanni, Milan, Italy

**Keywords:** Gender medicine, Sex-specific, Neonate, Preterm, Review

## Abstract

This narrative non-systematic review addresses the sex-specific differences observed both in prenatal period and, subsequently, in early childhood. Indeed, gender influences the type of birth and related complications. The risk of preterm birth, perinatal diseases, and differences on efficacy for pharmacological and non-pharmacological therapies, as well as prevention programs, will be evaluated. Although male newborns get more disadvantages, the physiological changes during growth and factors like social, demographic, and behavioural reverse this prevalence for some diseases. Therefore, given the primary role of genetics in gender differences, further studies specifically targeted neonatal sex-differences will be needed to streamline medical care and improve prevention programs.

## Introduction

The aim of gender medicine is to pursue accuracy and personalization of diagnosis and treatment based on gender evidences. Scientific studies, aimed at maximize the prevention programs, stress that there is a biological difference related to some diseases onset and to some drugs response. The difference by sex on neonatal mortality, calculated considering race and birth weight, was already known at the beginning of the last century [1], and it is inversely related to gestational age[2]. Recent data from the international database “Vermont Oxford Network” showed gender differences in both mortality and postnatal outcomes, with the worst prognosis for the male population [3]. Despite studies identifying sex as risk factor for some diseases in the last decades have progressively increased, such phenomenon is being understood only recently. This was possible analysing the development of organs and systems, as well as their functional recovery capacity following any injuries.

## Methods and materials

We reviewed the articles published around the last twenty years on PubMed/Medline (http://www.ncbi.nlm.nih.gov/PubMed), Embase (https://www.embase.com/search/quick) and Ovid (http://www.ovid.com/) using the following terms: gender medicine, sex, newborn, and preterm. Forty-seven articles meeting the criteria were included. Papers concerning diagnostic or surgical procedures related to congenital malformations, syndromes involving the sexual apparatus, and lesions of the sexual organs were excluded.

### Prenatal aspects and childbirth

Higher incidence of congenital diseases, preterm birth, and premature rupture of membranes have been observed in pregnancies with male newborns [4]. Women carrying male foetuses had higher rates of gestational diabetes mellitus, foetal macrosomia, failure to progress during the first and second stages of labor, cord prolapse, nuchal cord, and true umbilical cord knots. Higher incidence of caesarean sections (CS) and preterm births [5] with a higher overall mortality rate [6] have been found in male neonates [7]. The reasons are various and not fully understood yet.

A study on women undergoing elective CS without complications showed a higher pro-inflammatory response in the plasma of male infants subjected to lipopolysaccharide stimulation [8]. Such a result could be a reason for premature membranes’ rupture and might explain the different reaction to neonatal infections.

The rate of free beta-hCG is, in the first trimester, significantly higher in the female foetuses, while the contrary is for the pregnancy-associated plasma protein-A levels. That may explain an increased risk for Down syndrome reported in pregnancies with female foetuses, but without statistical significance [9]. Knippel et al. [10] found higher levels of ^alphafetoprotein^ (AFP) in male foetuses and, due to the higher incidence of malformations AFP-related in females, hypothesized a protective role for this protein.

A possible additional reason justifying the disadvantage of male birth is the increased metabolic request due to the acceleration of growth, which causes greater vulnerability to minimal reductions in fetal oxygenation and blood flow, during both pregnancy and labor. Such differences, albeit small in oxygenation and lactacidemia, could increase susceptibility to early neonatal infections [11], explaining the worst outcome in case of an adverse event.

### Drugs in pregnancy and postnatal effects

Antenatal drugs exposure causes different effects depending on sex. Neonatal abstinence syndrome secondary to prenatal opioid exposure is significantly more frequent and severe in the male population [12], while females show more benzodiazepine deficiency symptoms, either alone or combined with opioid [13].

Among opioid-exposed infants, antidepressant medication co-exposure is common. Some studies, addressed to investigate its long-term effects, reported a worse influence on male neonates, mainly resulted in an increasing length of hospitalization, but without statistical significance [14].

Infant gut microbiota, without differences between the sexes at birth but with small variations due to the type of delivery, can be influenced by maternal drugs assumption. In a group of newborns with mothers requiring anti-asthma treatments during pregnancy, the amount of Lactobacilli in faeces resulted significantly lower in males [15], while a higher value of Bacteroidacæ has been recorded in females.

### Postnatal period

postnatal adaptation is sex-related, due to both the higher prevalence of associated complications [16] and the ability to recover from an adverse event [17]. A neurobehavioral follow-up study on premature babies born at less than 28 weeks of gestational age found a lower incidence of complications such as cerebral palsy, deafness, blindness, and mental or psychomotor retardation in females [18].

Recently, a meta-analysis involving 41 studies and 625,680 neonates confirmed this theory and demonstrated greater clinical instability and need for invasive interventions in preterm males. Additionally, it reported higher rates of bronchopulmonary dysplasia (BPD), retinopathy of prematurity, necrotizing enterocolitis, intraventricular haemorrhage, and periventricular leukomalacia [19]. Although geographic factors, proper perinatal care and gestational age can reduce the gap between sexes, the feeling of generic “weakness” remains in males. Of note is its persistence even after hospital discharge, and especially for respiratory infections [20], until the first year of life [21].

Gender differences in the pulmonary develop are noticeable as early as 16–20 weeks of gestation. Mouth movements, related to both swallowing and intrauterine “respiratory function”, are more frequent in female fetuses [22]. Conversely, animal studies reported lower lung tissue stability [23], reduced gas exchange with no improvement in respiratory mechanics after steroid treatment [24], and an increased risk of lung injury due to hyperoxia [25] in males. A possible reason for this might be connected with the sex hormones levels circulating in the prenatal period. The amount of estrogen and progesterone is comparable between genders, as they both result from transplacental passage, but testosterone levels are higher in males [26]. Another hypothesis focuses on the alveolar epithelial transport of Na^+^ as a determinant of the perinatal pulmonary transition [27], with differences among the sexes. Finally, a study carried out by measuring the diversity in the expression of microRNA during fetal lung development hypothesized its role as a cofactor in lung diseases, both in neonatal and adulthood [28].

Furthermore, it is possible to hypothesize that delayed lung development observed in male newborns causes a gap between the development of the airways and the lung parenchyma, thereby increasing airway resistance. Overall, female fetuses produce surfactant earlier, move their mouths more, develop larger airways that are less reactive to insult, and develop more mature parenchyma. Therefore, males have a higher incidence and severity of respiratory distress syndrome, BPD, wheezing, asthma, and chronic diffuse interstitial lung disease, while cystic fibrosis is more severe in girls, who have a higher risk of complications and worse outcomes [29].

Recently a large and comprehensive systematic review and meta-analysis of preterm babies with persistent patent ductus arteriosus (PDA) showed no difference between boys and girls in both the incidence and the response rate to pharmacological treatment [30]. A common conception among neonatologists is the interaction between PDA and the respiratory evolution of the premature babies. The presence of a hemodynamically significant PDA is frequently suspected based on respiratory findings, such as increased oxygen or mechanical ventilation requirements. Although male gender is associated with an increased risk of RDS, higher rates of birth intubation, surfactant treatment, mechanical ventilation, and pneumothorax, the actual results suggest that the presence of PDA is unlikely to play a role in these sex differences in respiratory courses.

On the other hand, congenital heart disease (CHD) is significantly influenced by gender, not only in terms of incidence and severity, but also in postnatal evolution and long-term outcomes. However, this influence is not universal, and varies depending on the type of anomaly considered [31]. Females have a higher incidence of less serious CHD, such as interventricular and interatrial defects, pulmonary stenosis, and aortic coarctation, while major pathologies like Fallot tetralogy or left hypoplastic heart are more prevalent in males [32]. After surgical treatment, the volume index and ventricular masses are larger in males, as in the normal healthy population. Right ventricular hypertrophy and dilatation correlate with loading conditions in a similar way for both sexes. However, under comparable loading conditions, males show more severe functional impairment [33]. Although the clinical history of infants with CHD is related to gender, there is no significant prevalence for either sex. A higher mortality rate has been reported in older males with CHD, while sudden cardiac death is more prevalent in young males. However, mortality for CHD after surgery is higher among girls compared to boys, probably due to their smaller body size. Women are at higher risk of developing pulmonary arterial hypertension but at lower risk of adverse aortic outcomes, even though the possibility of them undergoing aortic surgery remains minimal. Moreover, females have a lower risk of infective endocarditis [34].

Observations from clinical research in humans have suggested a difference in brain and neuronal physiology based on sex differences that begin in the fetal and newborn period, and extend throughout the human lifespan into adulthood. In premature infants, girls have significantly lower cerebral blood flow (CBF) than boys of similar gestational and postnatal age [35]; however, adult females have higher CBF than males. The mechanism regulating these differences are not well-understood, but the relative immaturity of CBF auto-regulation in premature infants may be the reason why females with relatively lower cerebral blood flow have a lesser incidence of germinal matrix or intraventricular haemorrhage.

Pain sensitivity is another issue significantly connected with gender. Newborns and preterm male tolerate fewer painful stimuli [36, 37], although there is a difference in which side of the body is involved [38]. This is probably due to bilateral somatosensory cortical activation, which is less evident in females [39] and persists until adolescence [40]. Conversely, a study conducted on an ex-preterm cohort in adulthood showed a lower capacity to modulate pain in females with a consequent increased risk of developing persistent pathological pain, although the reason for this is still unclear [41].

Differences between sexes exist in cellular and molecular development [42], which affect both normal neuronal function and the effectiveness of various therapies [43] in cases of brain damage. However, the correlation with the behavioural and psychological aspects is still a matter of discussion [44].

Sexual dimorphism of the fetus manifests during pregnancy. Intrauterine and postnatal growth nomograms are sex-specific. There is increasing evidence showing that from fetal life, boys and girls have different responses to maternal nutrition, and that maternal breastmilk composition differs based on fetal sex [45]. Furthermore, early neonatal nutritional interventions affect boys and girls differently, and early nutrition has sex-specific effects on both body composition and neurodevelopmental outcomes [46, 47]. However, no studies have investigated whether nutritional requirements differ between the sexes. Thus, the current nutrition guidelines for preterm infants are unisex and could be sub-optimal. More information is needed to determine sex differences in infants’ macronutrient requirements, such as whether preterm females require higher fat intake and preterm males require higher protein intake for optimal growth and neurodevelopmental outcomes [48].

### Therapies effectiveness

Pharmacological treatments have varying efficacy and side effects depending on a patient’s sex, especially in preterm population [49]. Unfortunately, scientific literature seldom covers gender differences in infant pharmacology, whether in randomized controlled studies or meta-analyses.

The pharmacological inhibition of prostaglandin synthesis has been shown to promote the stability of germinal matrix vessels and prevent intraventricular haemorrhage (IVH) in preterm rabbit pups. A similar effect has been reported in humans. Two large North American trials investigated the early use of intravenous indomethacin in preterm infants at high risk for IVH, and the results showed a significant reduction in severe intraventricular haemorrhage in only the male papulation [50]. On the other hand, less positive long-term cognitive outcomes and a higher mortality rate were observed in female infants [51]. Therefore, this prophylaxis appears to be as beneficial for males as potentially harmful for females. Conversely, hydrocortisone for BPD prophylaxis is more effective in females, increasing bronchopulmonary dysplasia-free survival rate [52].

Experimental studies carried out by administering caffeine (an adenosine receptor antagonist) to rats have shown several positive effects on respiratory pattern, such as an increase in respiratory frequency in the early phase of response to hypoxia and in tidal volume in the late phase of response. This effect has been observed exclusively in male rats [53], probably due to the long-term effects on the nucleoside receptor system. In addition, the increased expression of the Adenosine (2 A) receptor, which is specific to male rats, may have affected adenosine-dopamine interactions that regulate chemosensory activity.

Therapeutic hypothermia is a widely used procedure to protect neonates from hypoxic–ischaemic brain injury [54], which was found to be more effective in the female population, particularly in medium and long-term outcomes [55]. For the same purpose, experimental treatment with the infusion of stem cells did not show differences between genders [56].

## Conclusions

The aim of gender medicine is to improve care by considering patient’s sex as a variable responsible for the onset and evolution of many diseases. Some differences are also reported among neonates, suggesting the need to consider sex variables in diagnostic and therapeutic pathways.

Overall, while the male population seems to be more affected by diseases and related complications in the first months of life, an inversion of trend has been noted during growth for some conditions (Table 1). In addition to genetic and physiological aspects, social, demographic, and behavioural factors may also play an important role in this tendency. Based on the findings of this paper, we believe that a systematic review, including more sources and a larger period, could better clarify the role of gender in neonatology. Therefore we believe it is necessary to carefully consider the sex variable in both scientific research and clinical practice to have the most appropriate approach towards patients and to apply the most suitable care, therapies, and prophylaxis.

**Table 1:** Summary of main differences in neonatal outcomes by gender

	Male	Female	RR	95% CI	Reference nr.
Preterm birth 28–37 wks	+	-	1.50	1.40–1.60	3, 5
Preterm birth < 28 wks	+	-	1.23	1.14–1.33	3, 5
Mortality within 12 h of birth	+	-	1.02	0.98–1.06	3, 4
Died before hospital discharge	+	-	1.15	1.12–1.17	3, 4
Neurobehavioral complications	+	-	2.00	1.60–1.50	18
Late onset sepsis	+	-	1.05	1.02–1.07	19
Transient tachypnea	+	-	1.25	1.03–1.52	20
Respiratory distress syndrome	+	-	1.09	1.04–1.14	19
Chronic lung disease	+	-	1.30	1.22–1.38	20, 29
Bronchopulmonary dysplasia	+	-	1.20	1.09–1.31	19, 20
Intraventricular haemorrhage	+	-	1.16	1.13–1.19	19
Retinopathy of prematurity	+	-	1.14	1.06–1.22	19
Necrotizing enterocolitis	+	-	1.14	1.03–1.26	19
Cystic fibrosis (mortality risk)	-	+	1.60	1.40–1.80	29
Mild congenital heart disease	-	+	1.23	1.19–1.27	32
Severe congenital heart disease	+	-	1.41	1.30–1.52	32
Pain related neonatal symptoms	+	-	1.14	1.05–1.23	38
Pain sensibility in ex-preterm	-	+	1.39	1.30–1.48	36, 37
Opioid abstinence syndrome	+	-	1.18	1.05–1.33	12
Benzodiazepine deficiency symptoms	-	+	1.51	1.04–2.21	13

**+** = more affected; **-** = less affected; **RR** = relative risk ratio; **95% CI** = 95% confidence intervals.

## Data Availability

Not applicable.

## Abbreviations

AFP: Alphafetoprotein
BPD: Bronchopulmonary dysplasia
CS: Caesarean section
CHD: Congenital heart disease
IVH: Intraventricular haemorrhage
